# Feature Extraction and Selection for Pain Recognition Using Peripheral Physiological Signals

**DOI:** 10.3389/fnins.2019.00437

**Published:** 2019-05-07

**Authors:** Evan Campbell, Angkoon Phinyomark, Erik Scheme

**Affiliations:** ^1^Department of Electrical and Computer Engineering, University of New Brunswick, Fredericton, NB, Canada; ^2^Institute of Biomedical Engineering, University of New Brunswick, Fredericton, NB, Canada

**Keywords:** affective computing, EMG, emotion recognition, feature extraction, feature selection, multimodal analysis, heat pain, physiological signals

## Abstract

In pattern recognition, the selection of appropriate features is paramount to both the performance and the robustness of the system. Over-reliance on machine learning-based feature selection methods can, therefore, be problematic; especially when conducted using small snapshots of data. The results of these methods, if adopted without proper interpretation, can lead to sub-optimal system design or worse, the abandonment of otherwise viable and important features. In this work, a deep exploration of pain-based emotion classification was conducted to better understand differences in the results of the related literature. In total, 155 different time domain and frequency domain features were explored, derived from electromyogram (EMG), skin conductance levels (SCL), and electrocardiogram (ECG) readings taken from the 85 subjects in response to heat-induced pain. To address the inconsistency in the optimal feature sets found in related works, an exhaustive and interpretable feature selection protocol was followed to obtain a generalizable feature set. Associations between features were then visualized using a topologically-informed chart, called Mapper, of this physiological feature space, including synthesis and comparison of results from previous literature. This topological feature chart was able to identify key sources of information that led to the formation of five main functional feature groups: signal amplitude and power, frequency information, nonlinear complexity, unique, and connecting. These functional groupings were used to extract further insight into observable autonomic responses to pain through a complementary statistical interaction analysis. From this chart, it was observed that EMG and SCL derived features could functionally replace those obtained from ECG. These insights motivate future work on novel sensing modalities, feature design, deep learning approaches, and dimensionality reduction techniques.

## 1. Introduction

Emotion is the basis of subjective experience that drives human behavior and regulates many physiological states. Throughout nearly all forms of life, motivation spurred by primal emotions like fear and desire facilitates successful adaptation to surrounding environments. In the field of affective computing, a desire to reciprocate this interaction has begun through emotion recognition and the development of affect sensitive systems. By monitoring these manifestations of emotion, called “affects,” an intelligent surrounding environment can respond to enhance engagement and cohesion with its participants. These systems are pervasive across applications such as pain detection (Nezam et al., 2018), education (Lara et al., 2018), workplace optimization (Zenonos et al., 2016), and more.

Systems have been constructed to respond to affective states; however, none have yet to register the full spectrum of emotions ubiquitously due to the sophistication and variability of emotions. Instead, affective computing has primarily embraced four theories that quantify affective state: expression, embodiment, neuroscience, and arousal-valance (Marsella et al., 2010). Specifically, expression outlines the relationship between affective states and their corresponding observable tendencies (Darwin, 1916). In contrast, embodiment characterizes emotions with their accompanying physiological symptoms (James, 1884). Dalgleish et al. (2009), and Borsook et al. (2010) demonstrated that many key cognition, emotion, and pain pathways are shared in the brain. Thus, a theory that concentrates on the brain as an indicator of affective state has shown merit in the application of existing techniques from affective neuroscience. Deconstruction of emotional states into the concepts of arousal and valence has facilitated the practical application of emotion classification (Russell, 1980; Lang et al., 1998). This two-dimensional scale has provided a framework for quantifying emotional state in many recent affective computing studies (Khalili and Moradi, 2009; Rahnuma et al., 2011 ;Zhang and Zhang, 2017).

The merits of each theory of affective state have been shown through a variety of experiments. The arousal-valance theory is used as a continuous-spectrum substitute for basic emotions, instead of using discrete categories. Arousal corresponds to the intensity of the emotion; whereas valance corresponds to the disposition (pleasant/unpleasant). For instance, as a response to harmful stimuli, pain can elicit a state of high arousal characterized by sympathetic arousal and heightened attention to the source of the stimuli. Alternatively, arousal and valance together can discriminate between complex emotions; both sadness and anger have negative valance, but anger has high arousal and sadness has low arousal (Shu et al., 2018). Within experiments, media materials are often used to evoke various responses on the arousal-valence curve that correspond to basic emotions (Zong and Chetouani, 2009). Developments in neuroscience have led to the use of functional magnetic resonance imaging (fMRI) (Han et al., 2015) and electroencephalogram (EEG) (Petrantonakis and Hadjileontiadis, 2010) in affective state studies. The theory of expression has been verified by recording facial features (Ekman and Freisen, 2003; Valstar and Pantic, 2012), posture (D'Mello and Graesser, 2009), and voice characteristics (Juslin and Scherer, 2005) in response to emotion-evoking material. Studies that use expression-based recognition, however, necessitate an isolated environment for audio monitoring, and privacy is a concern when monitoring video (Chen et al., 2018). An additional limitation of video is that individuals can consciously mimic or hide facial expressions confounding specificity and sensitivity, respectively.

With the advancement of wearable technologies, physiological signals can now be monitored non-intrusively, with high fidelity, and alleviate many of these privacy concerns. Studies that employ physiological signals such as electromyogram (EMG) (Wijsman et al., 2013), electrocardiogram (ECG)/ plethysmogram (PMG) (Valderas et al., 2015), skin conductance level (SCL, also known as galvanic skin response, GSR) (Murali et al., 2015), respiration rate (RR) (Wu et al., 2012), and pupil dilation (PD) (Babiker et al., 2013), have reinforced the embodiment theory of emotion by using physiological signals for emotion recognition. In addition to validating these theories, these studies corroborate the correlation between affective state and various modalities.

The emergence of multimodal studies has greatly enhanced the performance of affect-sensitive systems. While demonstrating superior performance, multimodal emotion recognition studies also provide a unique environment to contrast the unique and pooled discriminative ability of various physiological modalities. For example, EMG, ECG, SCL, and RR were used to mediate distractions during a daily commute using driver distress level (Healey and Picard, 2005). An overall accuracy of 97.4% was achieved in a three-level stress detection task with ECG and SCL contributing the most discriminative power. The use of SCL, PMG, PD, and skin temperature for the detection of stress induced by the Stroop Effect has also been explored (Zhai and Barreto, 2006), with classification accuracy of stress levels reaching 90.1%.

In order to achieve these high classification accuracies in emotion recognition, the selection of appropriate features may be considered as the most critical for machine learning. However, over-reliance on automated feature selection methods can be problematic, especially when conducted using small snapshots of data. The results of these methods, if adopted without proper interpretation, can lead to sub-optimal system design, or worse, the abandonment of otherwise viable and important features. To navigate this problem in a tangible way, we focus on a specific case study of emotion recognition; heat pain assessment, for which the selection of features and modalities is a current and acknowledged problem in the scientific literature.

The perception of pain is affected by emotion and the response to pain evokes an emotional response (Woo et al., 2015). While pain can be studied as a model for arousal, the development of pain recognition systems has considerable clinical relevance. Self-reported methods of reporting pain are subjective and thus ill-suited for the complexity of pain perception. Furthermore, self-reporting of pain is not always feasible for all patients, such as those experiencing trauma (Berthier et al., 1998), or those with dementia (Zwakhalen et al., 2006) or other cognitive impairments.

Many researchers have conducted experiments resulting in a wide range of performances in the classification of pain. A number of characteristics of these studies, however, have made their results difficult to interpret and harmonize. First, many have chosen to focus on a particular modality and/or feature type. Differing subsets of feature modalities (Werner et al., 2014; Kächele et al., 2017; Lopez-Martinez and Picard, 2017) or feature types (Chu et al., 2017) have detracted from the ability to translate and compare results between studies. Second, the absence of feature selection methods has failed to identify the source of enhancements compared to other studies (Chu et al., 2014). In others, some feature selection methods may have found an optimal feature set for a specific instance of a classification algorithm, but not necessarily for the generalizable classification problem (Walter et al., 2014; Gruss et al., 2015; Kächele et al., 2015). Third, subject-dependent feature selection protocols increase accuracy, but the source of improvement cannot be distinguished between subject specific information or classifier conformity to noise (Walter et al., 2014). These factors have led to a lack of consensus on the selection of optimal modalities and features for the classification of pain intensities. As more researchers build on the work of others, this could lead to the abandonment of otherwise useful modalities or the selection of sub-optimal features.

While the use of automated feature selection methods accelerates the discovery of meaningful techniques and features, their narrow scope can limit comprehension of the phenomena being classified. The field of automatic pain detection has indeed been streamlined by primarily focusing on classification accuracy, without necessarily emphasizing the underlying problem. Researchers have proposed a variety of methods for discriminating between perceived pain intensities given measured autonomous responses, yet no consensus has been reached due to a lack of comprehensive examination of their discriminative ability.

In this exploration, we aim to meet two purposes: (1) To obtain meaningful, discriminatory sets of generalizable features for pain recognition with minimal bias to the underlying methods selected. By conducting a one hundred epoch selection followed by a majority vote, the most frequently selected features are revealed. (2) To quantify associations between modalities and features to enhance comprehension of autonomic pain responses. By employing Mapper (Singh et al., 2007), a topological data analysis tool, an understanding of the unique and common discriminative aspects of available features is gained. With these newfound insights, differences between previous related works can be better understood and compared.

## 2. Materials and Methods

Categorization of pain levels was conducted using a pipeline, consisting of five stages: data collection, data pre-processing, feature extraction, feature selection, and classification. Briefly, this pipeline removed unwanted artifacts from the signals and segmented measurements into finite-length windows by pre-processing. The information density was increased in each of these windows by extracting features of relevant signal characteristics. These features were evaluated and screened to identify key discriminative features for use by the classifier. The classification algorithms then harnessed the information captured by these features to segregate the data into pre-defined classes of pain.

### 2.1. Emotion Data

The dataset—Biopotential and Video (BioVid) Heat Pain Database—outlined by Gruss et al. (2015) was adopted for this work as it was publicly available and includes physiological modalities common across a majority of related works. The collection of the data, as explained by Walter et al. (2013), was carried out in accordance with the recommendations of the ethics committee of the University of Ulm with written informed consent from all subjects. All subjects gave written informed consent in accordance with the Declaration of Helsinki. The protocol was approved by the ethics committee of the University of Ulm (196/10-UBB/bal). The data include surface EMG from the zygomaticus (zEMG), corrugator (cEMG), and trapezius (tEMG) muscles, ECG, and SCL elicited in response to a pain-inducing heat stimulus. For each trial, a randomly selected pain stimulus level was applied for 4 s and the physiological response was recorded for 5.5 s. Eighty-five participants conducted 20 trials at each of the five pain-intensity levels.

Specifically, heat-pain stimuli levels were calibrated on a per subject basis. A *baseline*, (*B*), was defined as 32°C. The first pain level, *pain threshold* (*T1*), was given as the transition from a sensation of warmth to a burning, pulling, or stretching sensation. The fourth and highest pain level, *pain tolerance* (*T4*), was given by the upper limit of tolerable pain due to heat. Two linearly spaced values between pain threshold and pain tolerance, *T2* and *T3*, provided additional resolution of intensity levels, but were not tied to a sensory trigger event. For simplification, detection of these heat-pain levels were divided into four classification problems, (1) pain threshold problem (*B vs. T1*), (2) pain tolerance problem (*B vs. T4*), (3) three-class problem (*B vs. T1 vs. T4*), and (4) five-class problem (*B vs. T1 vs. T2 vs. T3 vs. T4*).

### 2.2. Data Pre-processing

Prior to feature extraction, the physiological signals underwent pre-processing to remove unwanted artifacts. The raw EMG and ECG signals were bandpass filtered using 4th order Butterworth filters with pass bands of 20–250 Hz and 0.1–250 Hz for EMG and ECG, respectively (Walter et al., 2014). The signals were then broken down into their intrinsic mode functions by use of empirical mode decomposition (EMD) (Huang et al., 1998). This enabled the intrinsic mode function that corresponded to power-line interference to be removed from the measured signals. Afterwards, the Hilbert spectrum was used to highlight EMG activity for data segmentation (Azarbad et al., 2014). Finally, features were normalized by z-score, zero-mean unit-variance distribution, to enforce scale across modalities and feature types.

### 2.3. Feature Extraction

Feature extraction is the process of increasing information density by retrieving key properties from a larger element of data. These properties are leveraged to build models able to predict the class of sampled data. Within this context, features were extracted from physiological data during the 5.5 s window after the onset of the painful stimuli. Largely, feature domains are classified by the continuum from which they were calculated, i.e., time domain and frequency domain. Time domain features extract information directly from the sampled time series after pre-processing. SCL time domain features have been found to be effective in arousal quantification, where responses to stimuli were shown to be largely time-invariant (Bach et al., 2010). Observation of EMG time domain reveals non-stationarity (Lei et al., 2001). Regardless, time domain EMG features have been shown to yield impressive accuracies in controlled settings (Phinyomark et al., 2013). In contrast, frequency domain features are calculated from transformed data and involve characterization of the spectral domain. The phasic component of SCL, the skin conductance response, has also shown to be correlated with arousal (Cuthbert et al., 2000; Bradley and Lang, 2003). Alternatively, signals that have a characteristic profile like ECG may require the use of a transform to identify landmarks of the signal as features. For instance, heart rate variability metrics extracted from ECG have been used to measure autonomic nervous system activity (Jiang et al., 2017).

In addition to these feature domains, feature extraction methods may also be categorized by the theoretical type of information they are designed to extract. Within this study, several *theoretical* feature types were explored, including those that capture (1) signal amplitude, (2) variability, (3) stationarity, (4) entropy, (5) linearity, (6) similarity, and (7) frequency properties (Gruss et al., 2015). A full list of these 155 features is shown in Table 1. Abbreviations of feature names were chosen to be as concise as possible while still translating across the related physiology literature. The mathematical definitions of these features can be found within the works listed in the definition column. The zEMG, cEMG, and tEMG modalities were each characterized by 39 features (#1-#39), the SCL modality was characterized by 35 features (#1-#35), and the ECG modality was characterized by three features (#40-#42) (Table 1).

**Table 1:** List of all features included in the exploration, in alphabetical order and theoretical groups.

**#**	**Abbreviation**	**Theoretical group**	**Full name**	**References**
1	HOMAV1	Amplitude	First Higher-Order Mean Absolute Value	Phinyomark et al., 2014
2	HOMAV1n	Amplitude	Normalized 1st Higher-Order Mean Absolute Value	Phinyomark et al., 2014
3	HOMAV2	Amplitude	Second Higher-Order Mean Absolute Value	Phinyomark et al., 2014
4	HOMAV2n	Amplitude	Normalized 2nd Higher-Order Mean Absolute Value	Phinyomark et al., 2014
5	MAV	Amplitude	Mean Absolute Value	Phinyomark et al., 2012
6	P2P	Amplitude	Peak to Peak Amplitude	Walter et al., 2014
7	PK	Amplitude	Peak Amplitude	Walter et al., 2014
8	RMS	Amplitude	Root Mean Square	Phinyomark et al., 2012
9	TMNP	Amplitude	Mean Relative Time of the Peaks	Phinyomark and Scheme, 2018
10	TMNV	Amplitude	Mean Relative Time of the Valleys	Phinyomark and Scheme, 2018
11	IQR	Variability	Interquartile Range	Walter et al., 2014
12	R	Variability	Range	Walter et al., 2014
13	SD	Variability	Standard Deviation	Walter et al., 2014
14	VAR	Variability	Variance	Phinyomark et al., 2012
15	IDS	Stationarity	Interal Degree of Stationarity	Cao and Slobounov, 2011
16	MD	Stationarity	Median	Walter et al., 2014
17	MIDS	Stationarity	Modified Integral Degree of Stationarity	Cao and Slobounov, 2011
18	MMNDS	Stationarity	Modified Mean Degree of Stationarity	Cao and Slobounov, 2011
19	SDMN	Stationarity	Standard Deviation of Mean Vector	Walter et al., 2014
20	SDSD	Stationarity	Standard Deviation of Standard Deviation Vector	Walter et al., 2014
21	ApEn	Entropy	Approximate Entropy	Ferenets et al., 2006
22	FuzzyEn	Entropy	Fuzzy Entropy	Al-sharhan et al., 2001
23	SampEn	Entropy	Sample Entropy	Richman and Moorman, 2000
24	ShannonEn	Entropy	Shannon Entropy	Ferenets et al., 2006
25	SpectralEn	Entropy	Spectral Entropy	Ferenets et al., 2006
26	LDF	Linearity	Lag Dependence Function	Walter et al., 2014
27	PLDF	Linearity	Population Lag Dependence Function	Walter et al., 2014
28	CC	Similarity	Correlation Coefficient	Kennedy, 2007
29	MDCOH	Similarity	Median Coherence	Dukic et al., 2017
30	MI	Similarity	Mutual Information	Chen et al., 2003
31	MICOH	Similarity	Modified Integral of Coherence	Dukic et al., 2017
32	MNCOH	Similarity	Mean Coherence	Dukic et al., 2017
33	MMNCOH	Similarity	Modified Mean Coherence	Dukic et al., 2017
34	BW	Frequency	Bandwidth	Walter et al., 2014
35	CF	Frequency	Center Frequency	Walter et al., 2014
36	MDF	Frequency	Median Frequency	Phinyomark et al., 2012
37	MNF	Frequency	Mean Frequency	Phinyomark et al., 2012
38	MOF	Frequency	Mode Frequency	Walter et al., 2014
39	ZC	Frequency	Zero Crossings	Phinyomark et al., 2012
40	MNRR	Variability	Mean Resting Rate	Shaffer and Ginsberg, 2017
41	RMSSD	Variability	Root Mean Square Successive Interval Differences	Shaffer and Ginsberg, 2017
42	slopeRR	Variability	Slope Resting Rate	Shaffer and Ginsberg, 2017

*Feature abbreviations are included along with theoretical feature types and an accompanying article with mathematical definition*.

### 2.4. Feature Selection

Feature selection is the process of determining a *subset* of features that provide meaningful information to the classification problem. In contrast to feature extraction, which improves information density, feature selection aims to improve quality with a minimum loss of information. Machine learning-based feature selection involves ordering a set of features based on some criterion, such as discriminative power. Once ranked, features are incrementally and iteratively added to the classification model until some desired threshold was met.

**Figure 1:**
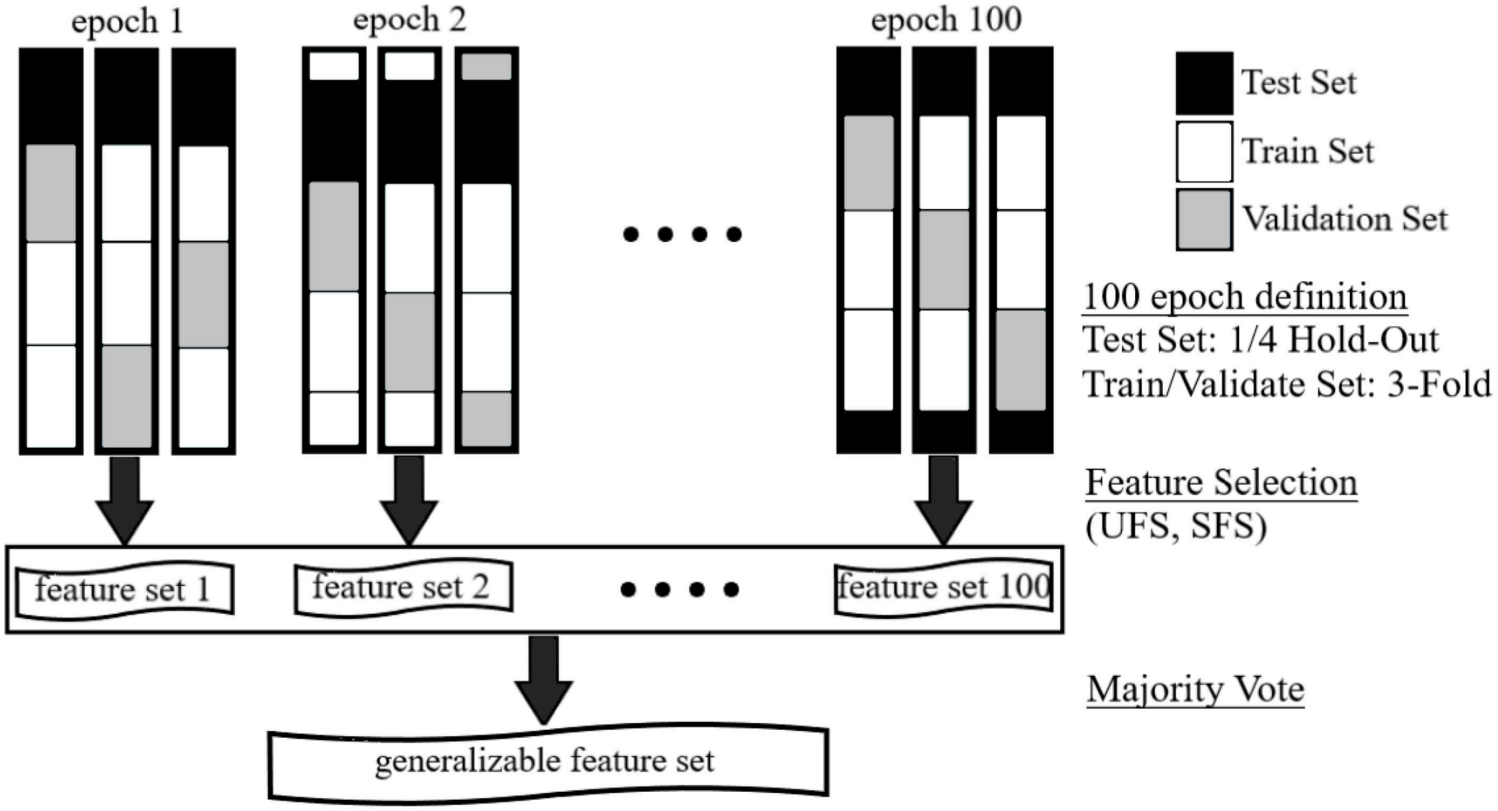
Illustration of one-hundred-epoch hold-out-and-*k*-fold cross-validation scheme used for machine learning-based feature selection approaches.

To improve the robustness of the resulting feature sets and reduce the variations observed in the results of previous studies, a one-hundred-epoch hold-out-and-*k*-fold cross-validation (CV) scheme was employed (Figure 1). Specifically, 100 independently and randomly generated subsets of the dataset were segmented to provide the classifier with a wide range of classification tasks. Data were stratified on the basis of subject and heat-pain level to ensure constant representation of subjects and stimulus level across all CVs. In each of the epochs, a one-quarter hold-out was used to ensure that a test set remained unseen during the selection process. In this way, 75% of each CV was used as a training set, while the remaining 25% were reserved as a test set. Each training set was further divided into a 3-fold CV. Two of the folds (50% partition of the original dataset) were used as a training set for feature selection process and the third (25% of the original dataset) was used as a validation set. These resulted in 10 and 5 samples per subject per stimulus level, respectively. An illustration that outlines the stages of the one-hundred-epoch cross-validation feature selection scheme is shown in Figure 1.

Feature selection was conducted using commonly employed feature selection approaches: univariate feature selection (UFS) and sequential forward selection (SFS). First, UFS involves the characterization of features, quantifying their discriminative power using a univariate statistical test such as an analysis of variance (ANOVA) *F*-value, distance correlation coefficient, or mutual information. For instance, Walter et al. (2014) previously ranked a set of features derived from this dataset based on their *p*-value. Within this study, our approach for UFS used *F*-value as the criterion for filter-based feature selection. *F*-value is the ratio of variance described by the feature to the variance not explained by the feature. The *F*-value for each feature was determined through a one-way ANOVA. The features were then ranked according to their mean *F*-value determined from the feature selection train set of each CV.

Second, SFS involves the direct use of a classifier in the determination of the most beneficial features for inclusion. The classifier is used as an objective function to judge the classification performance of features. For instance, Kächele et al. (2015) uses a similar wrapper-based feature selection using the classification accuracy of a support vector machine (SVM) algorithm. Within this study, our approach for SFS used a Naïve Bayes classifier employed as the objective function to ensure features that had higher independence from included features and improve model accuracy were prioritized. The process begins by selecting the one feature that yields the highest individual classification performance. All remaining features are then evaluated in conjunction with this feature, and the feature that adds the most discriminative power is added. The process is then repeated iteratively using the remaining set of available features until all features have been added or until no significant improvement is gained by the addition of more features.

### 2.5. Classification

These machine learning-based feature selection protocols were repeated for each of the four classification problems (i.e., pain threshold problem, pain tolerance problem, three-class problem, and the five-class problem). After feature selection process was completed and robust feature sets were determined, an SVM classifier was constructed for each training set and tested against the corresponding test set. The classification stage was conducted using a set of SVMs with linear kernels in a one-against-one strategy.

The number of features included in the robust set for both UFS and SFS were determined *post hoc* by two methods: *local maximum* and *global maximum*. First, the set yielding the first local maximum classification accuracy was defined as the number of features included where adding the next feature yielded no significant or substantive difference in classification accuracy (*p* < 0.05). For each CV, a local maximum was identified. The average local maximum across all CVs dictated the number of features included in the robust feature set. Identification of maxima are especially important for classification since, unlike regression problems, the addition of ambiguous feature may hinder both computation time and accuracy. Second, the sets of features that yielded the global maximum classification accuracy, indicating an upper performance limit, were identified. These global maxima were determined as the mean number of included features that yielded the maximum accuracy across all CVs. This threshold occurred when many more features were included than feasible in any practical application; however, this metric was useful in understanding the variance in feature information across the entire dataset and the measure of complexity of the classification problem. For example, global maxima that occurred with a small number of features correspond to low variance across features in the dataset; whereas, global maxima that occurred with a large number of features correspond to broad range of feature information across all features.

### 2.6. Topological Data Analysis

In addition to the machine learning-based feature selection approach, a cluster analysis tool based on topological data analysis (TDA) was employed to highlight associations between feature types. A topological simplification approach called Mapper (Singh et al., 2007) produced controlled simplifications and visualizations based on similarity or metric characteristics of the dataset. The controlled simplification comprised of a network that grouped complex high-dimensional data into a lower dimensional projection while preserving the associations present in the high-dimensional state. It is expected that features that have similar definitions characterize the same information and are thus grouped together. The intent of this algorithm, however, was to identify sets of features across modalities and feature types that characterize similar information for novel insight (Phinyomark et al., 2017). The use of Mapper as a cluster analysis tool has shown success in identifying an unknown subtype of breast cancer (Nicolau et al., 2011), analyzing the organization of the brain while processing complex tasks (Saggar et al., 2018), and has been validated on several datasets including genomic and spinal cord injury data (Lum et al., 2013).

#### 2.6.1. The Mapper Algorithm

The process required to form these simplifications consists of a pipeline of four stages:

1. *Transforming raw data into a point cloud*: the global shape of high-dimensional feature data was extracted and represented as a point cloud of data (low-dimensional) using a distance matrix (Euclidean distance, in this study). The 155 features comprised the rows of the matrix, and the 8,500 feature values (20 trials × 5 pain levels × 85 subjects) comprised the column of the matrix.
2. *Segmenting the point cloud data into overlapping regions using a filter function*: To analyze the similarity between features, the distance to the *k*th nearest neighbor (*k*-NN) (*k* = 2), an (inverse) measure of density, was employed as a filter function. The *resolution* of the network was determined by defining a set of regions that span the entire domain of the filtered dataset. This set contains *N* regions which overlap one another by *L*%. In the case of this study, a network was defined as a consistent structure reproducible across multiple CVs, and was found using *N* and *L* of 4 and 50%, respectively. It should be noted that the application of a single filter function allowed for the data to be transformed from its original high-dimension to one-dimensional projection. Multiple filter functions can be used at a time to create a multidimensional network; however, this added complexity was not necessary for this study.
3. *Applying a clustering approach to create clusters from each region*: the type of clustering that was used within this study was hierarchical cluster analysis with Ward's minimum variance method (Ward, 1963). Each of the clusters that result from this process served as a node in the topological network.
4. *Constructing the topological network*: nodes were connected to one another with an edge when sets of nodes contained the same features. The edge width was based on the amount of shared features between them. As a result, a topological feature chart was created.

For an extended coverage of a time-series TDA processing pipeline, the reader is encouraged to consult Phinyomark et al. (2018).

#### 2.6.2. Interpretation of Topological Networks

The relationship depicted by the topological network is specific to the filter function applied in the first stage of Mapper. By classifying the features by their smallest pairwise Euclidean distances, the similarity between features was quantified. The nodes of the topological network can be considered as functional groups of emotion features. Features that are grouped together within nodes located at small *k*-NN distances express very similar information (as with the nodes near the left edge of Figure 4). The information characterized by such a node can therefore be represented using a very small number of its features. On the other hand, although features with high *k*-NN distance (more independent features) can be locally grouped into clusters, these features contain less similar information (as with the nodes near the right edge of Figure 4). The number of features required to describe the information contained in these nodes is much higher, by comparison.

The shape of the resulting topological network greatly depends on the resolution defined in the second stage of Mapper. With larger *N* and *L*, the network is more sensitive to fine details in its structure. Conversely, by decreasing these parameters, the network is more sensitive to coarse details in its structure. Conventionally these parameters are chosen by manually tuning these parameters to get a stable network.

Once the network was rendered, additional cues were used to provide more insight into the contents of each node. The number of features within the node was indicated by an Arabic numeral within the node and the node size was scaled accordingly (Figures 3, 4). The nodes were also divided and colored according to their composition of modality (Figures 3, 4). At a glance, general information, like features grouped according to modality, can be extracted from this network; however, to extract more specific information topological feature charts must be employed. For an extended coverage of how to interpret a topologically informed chart of feature space, the reader is encouraged to consult Phinyomark et al. (2017).

#### 2.6.3. Complementary Feature Sets

In this work, the topological feature chart tool was also used to investigate and explain the feature sets determined here, as well as those proposed by previous studies using the BioVid database. For readability, the feature sets from these studies are abbreviated as FS1, FS2, FS3, and FS4 for Walter et al. (2014), Kächele et al. (2015), Kächele et al. (2017), and Gruss et al. (2015), respectively, as outlined as follows:

- *FS1* (3 features): cP2P, cShannonEn, hslopeRR.
- *FS2* (26 features): sSDSD, cP2P, cPK, cR, cSDSD, cSD, cRMS, cMAV, cHOMAV1, cTMNV, cTMNP, cHOMAV2, zPK, sVAR, cVAR, zP2P, zR, cIQR, sApEn, cCF, sR, sFuzzyEn, sP2P, zSDSD, zVAR, sHOMAV2n.
- *FS3* (5 features): sSDSD, tP2P, hslopeRR, tPK, tZC.
- *FS4* (10 features): zCC. zSDMN, cPK, cCC, cMI, tCC, zRMS, zLDF, zVAR, tMI.

### 2.7. Statistical Analysis

To generalize feature and functional group relationships present within this analysis, the distributions of the baseline and pain tolerance conditions were tested for statistical and substantive difference. First, a linear model was formed to isolate the feature response to pain from inter-subject variation. Normality of the residuals were determined using the Kolmogorov-Smirnov test. Wilcoxon rank sum tests were then used in the case of a non-normally distributed residual feature vector; whereas *t*-tests were used when the residual feature vector was normally distributed. Substantive significance was used to compliment statistical significance when applicable. The Cohen's effect size, *d*, was used to quantify the substantive significance into three categories: small, medium, and large categories for effect sizes of 0.2, 0.5, and 0.8, respectively (Cohen, 1988). These coefficients were used to determine the observable relationship between the included physiological modalities driven by the autonomic nervous system (ANS) and the heat-pain level grouped by the functional information they capture.

## 3. Results

The selection and classification results of the two feature selection processes, UFS and SFS, are shown in Tables 2, 3. The first local maximum classification accuracies that were found using the top ranked features for pain threshold, pain tolerance, three-class, and five-class problems were 3, 1, 2, and 3 using UFS, and 5, 8, 8, and 4 using SFS, respectively. Additionally, global maxima were found at 36, 44, 49, and 47 features for UFS and 38, 78, 65, and 73 for SFS. From these details, the feature sets determined during SFS for each task were labeled as FSa, FSb, FSc, and FSd for pain threshold, pain tolerance, three-class, and five-class problems, respectively.

- *FSa*: cCC, tCC, zCC, cHOMAV2n, cShannonEn.
- *FSb*: cCC, cRMS, tCC, tMAV, sSDSD, zCC, zIQR, cP2P.
- *FSc*: cCC, cPK, tCC, sSDSD, hslopeRR, zCC, cR, cShannonEn.
- *FSd*: cP2P, cCC, tCC, sSDSD.

**Table 2:** Classification performance of features using UFS.

***i***	**Pain threshold**			**Pain tolerance**			**Three-class**			**Five-class**		
	**Feature**	***F*-Value**	**Accuracy (%)**	**Feature**	***F*-Value**	**Accuracy (%)**	**Feature**	***F*-Value**	**Accuracy (%)**	**Feature**	***F*-Value**	**Accuracy (%)**
1	cCC	547.52	72.31 ± 1.27	**cR**	**702.81**	**72.86** **±** **1.12**	cCC	536.47	49.64 ± 0.87	cCC	444.48	28.81 ± 0.65
2	tCC	484.56	78.37 ± 1.15	cP2P	702.41	72.89 ± 1.12	**cR**	**518.41**	**63.22** **±** **1.14**	tCC	383.7	32.19 ± 0.68
3	**zCC**	**210.86**	**79.38** **±** **1.18**	cPK	650.11	72.72 ± 1.14	cP2P	517.96	63.22 ± 1.13	**cR**	**294.41**	**40.62** **±** **0.7**
4	tMICOH	155.25	79.27 ± 1.19	cCC	640.95	84.43 ± 0.96	cPK	479.09	63.08 ± 1.16	cP2P	294.22	40.62 ± 0.69
5	tMNCOH	155.25	79.28 ± 1.19	cSD	617.44	84.44 ± 0.89	tCC	456.92	67.04 ± 1.02	cPK	278.13	40.68 ± 0.76
6	cMNCOH	113.4	79.21 ± 1.18	cRMS	617.15	84.45 ± 0.89	cSD	445.75	66.91 ± 0.96	cSD	266.09	40.52 ± 0.79
7	cMICOH	112.78	79.22 ± 1.18	cSDSD	609.82	84.47 ± 0.9	cRMS	445.32	66.91 ± 0.96	cRMS	265.88	40.51 ± 0.8
8	zMICOH	49.2	79.18 ± 1.23	cMAV	555.3	84.73 ± 0.95	cSDSD	445.18	66.9 ± 0.98	cSDSD	261.74	40.3 ± 0.82
9	zMNCOH	49	79.21 ± 1.2	cTMNV	554.45	84.71 ± 0.94	cMAV	401.88	66.83 ± 0.98	cMAV	245.97	40.47 ± 0.8
10	sMICOH	13.29	79.11 ± 1.17	cTMNP	545.99	84.6 ± 0.94	cTMNV	400.05	66.73 ± 0.96	cTMNV	239.48	40.49 ± 0.78
36	***tSDME***	***4.33***	***80.38±1.13***	sR	236.66	90.6 ± 0.85	zZC	162.89	70.22 ± 0.96	sR	92.06	43.2 ± 0.8
44	cBW	3.15	80.2 ± 1.2	***cMNCOH***	***193.51***	***90.96±0.8***	cMNCOH	113.11	70.64 ± 1.01	sSD	68.58	43.1 ± 0.84
47	zR	2.89	80.25 ± 1.2	cMDF	151.55	90.82 ± 0.78	hRMSSD	95.12	70.52 ± 1	***hRMSSD***	***48.89***	***43.32±0.79***
49	cFuzzyEn	2.81	80.3 ± 1.18	cMNF	149.31	90.91 ± 0.73	***tPK***	***88.13***	***70.83±1.06***	sMICOH	47.89	43.27 ± 0.78

*i denotes the rank of feature's F-value and SFS iteration included in the model. Feature abbreviation, and the classification accuracy (mean ± sd) were displayed for each of the four classification problems. Local maxima are indicated by bolded entries in the upper part of table. Iterations were abridged within the lower part of table where global maxima for each classification task are indicated by bold entries*.

**Table 3:** Classification performance of the three most frequently selected features for each SFS iteration.

***i***	***R***	**Pain threshold**			**Pain tolerance**			**Three-class**			**Five-class**		
		**Feature**	**Votes (%)**	**Accuracy (%)**	**Feature**	**Votes (%)**	**Accuracy (%)**	**Feature**	**Votes (%)**	**Accuracy (%)**	**Feature**	**Votes (%)**	**Accuracy (%)**
1	1	cCC	100	72.3	cCC	100	73.7	cCC	92	48.6	cP2P	61	29.9
	2	–	–	–	–	–	–	cP2P	5	48.4	cR	13	29.9
	3	–	–	–	–	–	–	cR	3	48.3	cCC	13	29.7
	E	–	–	–	–	–	–	–	–	–	Other	13	29.5-29.8
2	1	tCC	100	78.4	cRMS	47	83.8	cPK	61	62.7	cCC	87	38.4
	2	–	–	–	cP2P	19	84.0	cR	19	62.9	cPK	7	35.5
	3	–	–	–	cSD	16	84.0	cP2P	12	63.0	cP2P	4	33.3
	E	–	–	–	E	18	83.4	cCC	8	61.6	cR	2	35.4
3	1	zCC	84	79.3	tCC	100	86.2	tCC	100	66.6	tCC	100	40.6
	2	cHOMAV1	4	79.0	–	–	–	–	–	–	–	–	–
	3	sSDSD	2	78.8	–	–	–	–	–	–	–	–	–
	E	Other	10	77.4-78.7	–	–	–	–	–	–	–	–	–
4	1	cHOMAV2n	17	79.8	tMAV	28	87.8	sSDSD	91	68.1	**sSDSD**	**48**	**41.4**
	2	cShannonEn	10	79.8	sSDSD	17	87.5	hslopeRR	5	67.7	sApEn	12	41.2
	3	cHOMAV1n	10	79.9	tRMS	16	87.3	sP2P	2	67.5	cShannonEn	7	41.1
	E	Other	63	78.5-79.2	Other	39	87.3-88.1	–	–	–	Other	33	40.6-41.1
5	1	**cShannonEn**	**7**	**80.3**	sSDSD	32	89.0	hslopeRR	72	69.0	hslopeRR	28	41.5
	2	zPK	6	80.1	zCC	16	88.7	sSDSD	6	68.9	sSDSD	17	41.8
	3	sMICOH	5	80.0	cApEn	7	88.4	tRMS	4	68.9	sApEn	8	41.9
	E	Other	82	78.7-79.8	Other	45	87.6-89.2	Other	8	68.0-68.1	Other	47	41.2-42.1
6	1	sMICOH	6	80.4	zCC	55	89.4	zCC	61	69.5	tCC	20	42.0
	2	zShannonEn	5	80.4	cApEn	4	89.3	cShannonEn	15	69.5	zIQR	11	41.9
	3	cPK	4	80.3	sSDSD	3	89.2	tShannonEn	7	69.4	hslopeRR	7	42.0
	E	Other	85	78.8-80.2	Other	38	87.7-89.9	Other	17	68.2-68.8	Other	62	41.4-43.0
7	1	sMICOH	4	80.6	zIQR	21	89.9	zCC	17	69.9	zCC	20	42.5
	2	cIQR	4	80.4	zCC	12	89.8	cShannonEn	10	70.0	cShannonEn	10	42.4
	3	zMAV	4	80.3	zVAR	10	89.8	cSampEnz	7	70.0	zIQR	9	42.4
	E	Other	88	79.0-80.2	Other	57	87.7-90.2	Other	66	68.3-69.5	Other	61	41.8-43.2
8	1	zPLDF	5	80.0	**zPLDF**	**6**	**90.3**	**tSDSD**	**8**	**70.3**	zCC	18	43.0
	2	sIDS	4	80.0	tMIDS	4	90.2	zIQR	7	70.3	zIQR	7	42.9
	3	cShannonEn	3	80.1	zMIDS	4	90.1	cFuzzyEn	6	70.3	sVAR	6	42.9
	E	Other	88	79.2-80.6	Other	86	88.5-90.7	Other	79	68.6-69.6	Other	69	41.7-43.4
**Classification problem**							***i***					**Accuracy(%)**	
Pain threshold							38					80.8 ± 1.1	
Pain tolerance							78					90.9 ± 0.9	
Three-class							65					71.1 ± 1.1	
Five-class							73					43.3 ± 0.8	

*i denotes the iteration of the feature selection protocol. R denotes the rank of the feature determined through majority vote. Feature abbreviation, the number of CVs the feature appears across (Votes), and classification accuracy (Accuracy) are displayed for each of the four classification problems. – denotes that the top 3 features or 100 votes were already allocated. Where the top 3 features did not inlcude all CV votes, remaining results are summarized with accuracy ranges as “Other”. Bolded entries indicate that a local maxima was reached for the classification problem. Global maxima determined for all classification tasks using the SFS protocol are abridged in the bottom table. Number of features for the global maxima were included as i with corresponding means and standard deviations of accuracies across CVs given in percentages*.

**Figure 2:**
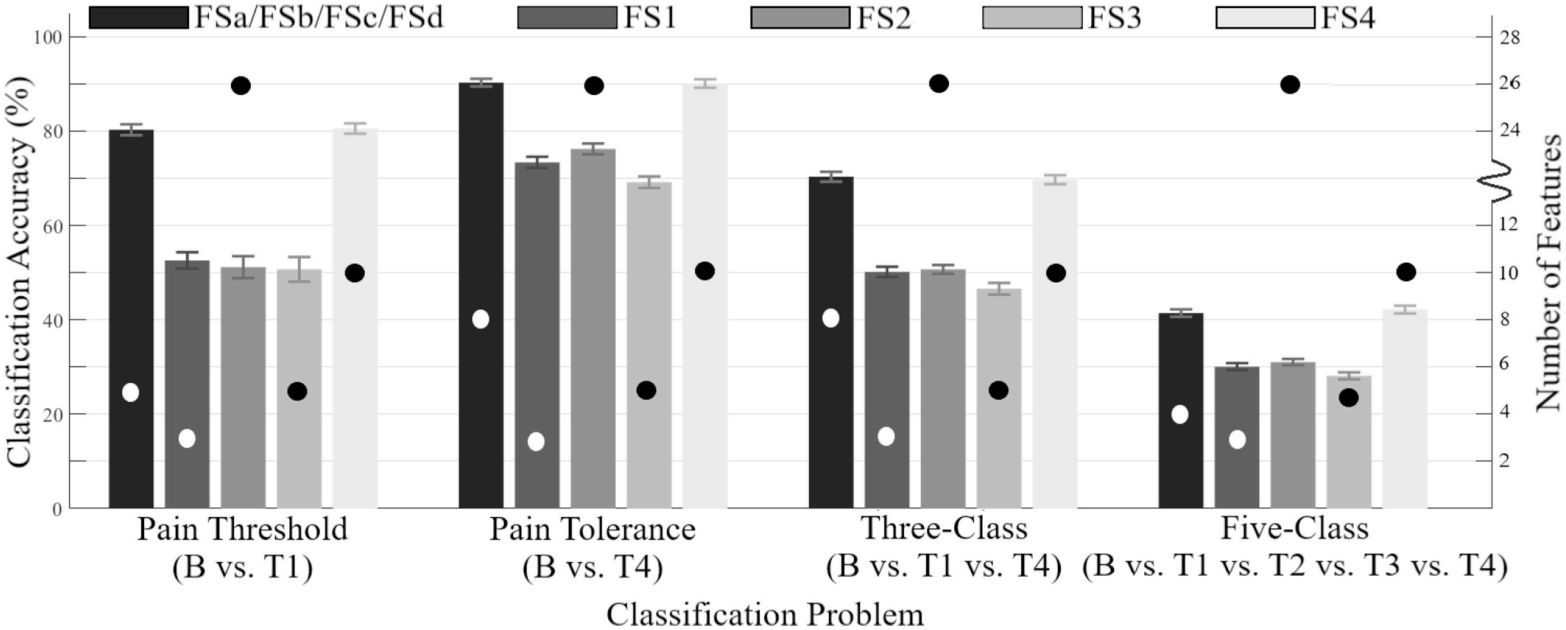
Accuracy of feature sets across all 100 CVs determined by SFS (FSa-FSd) as compared to those previously identified in the literature (FS1-FS4) (y-axis on the left side). Error bars are representative of standard deviation across all CVs. Circles indicate the number of features within each feature set (y-axis on the right side).

The performance of the robust feature sets determined by SFS were directly compared to the previous feature sets from the literature (FS1-FS4), as shown in Figure 2. The proposed sets were found to be a significant improvement compared to the state-of-the-art configuration. By achieving accuracies consistent with previously described state-of-the-art feature sets while using fewer features, the generalizability and computational costs of the system have are improved (Dietrich et al., 1999).

**Figure 3:**
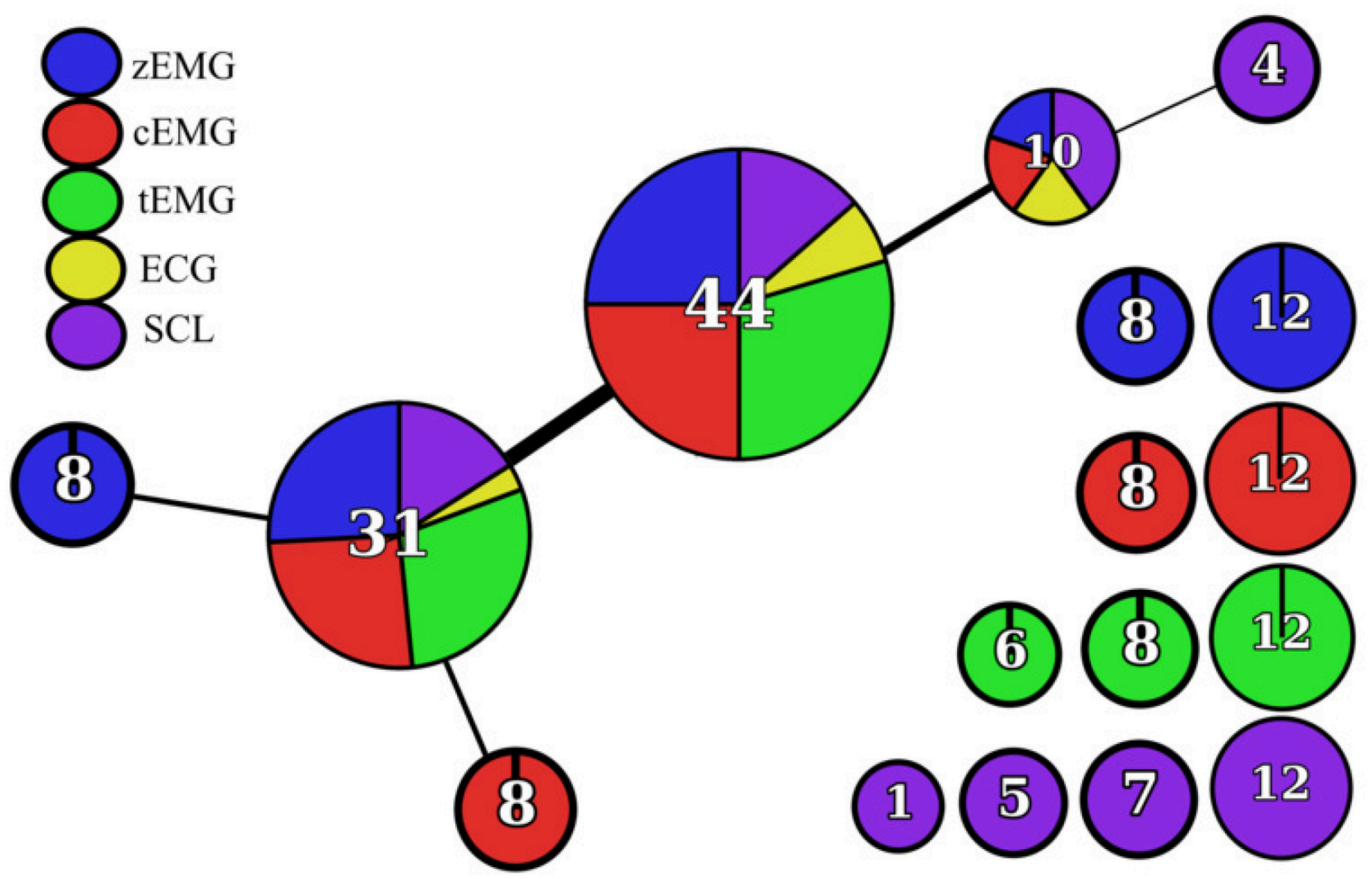
Topological network rendered by the Mapper algorithm using *k*-NN distance as the filter function.

**Figure 4:**
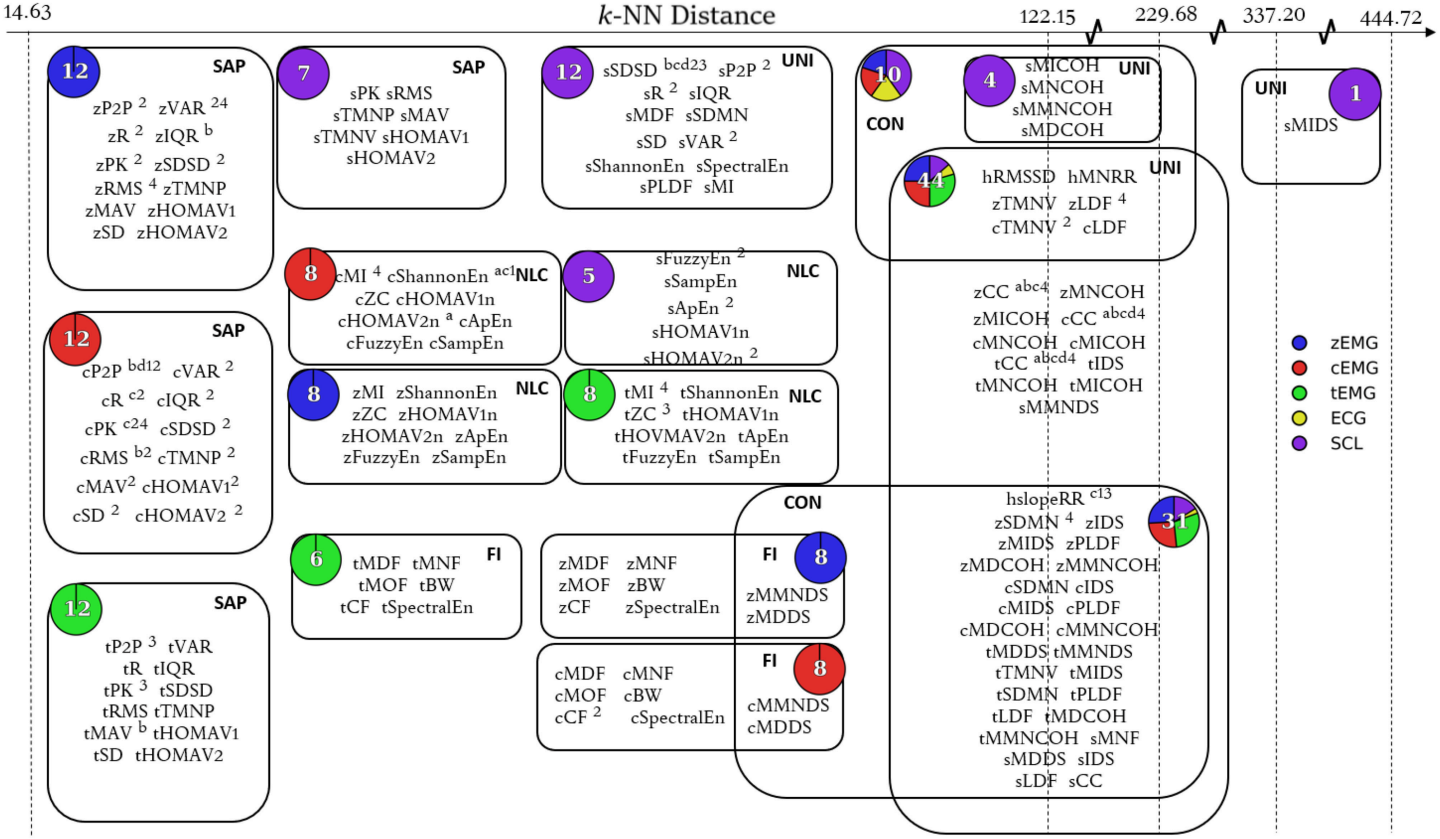
Topological feature chart. Node expansion highlighting key features selected within the SFS protocols for all classification tasks (FSa-FSd) and feature sets identified through other works of literature (FS1-FS4). Features belonging to defined feature sets were identified through superscripts (FSa: ^*a*^, FSb: ^*b*^, FSc: ^*c*^, FSd: ^*d*^, FS1: ^1^, FS2: ^2^, FS3: ^3^, FS4: ^4^). Node composition by modality is shown by pie charts. Node functional group is denoted by bold acronym (SAP, Signal Amplitude and Power; NLC, Nonlinear Complexity; FI, Frequency Information; UNI, Unique; CON, Connecting).

The empirical design of the feature sets were also assessed using the Mapper approach. Figure 3 shows the corresponding network based on the *k*-NN distance between features. Figure 4 consists of a topological feature chart that explicitly displays and contrasts the features selected here (FSa-FSd) and in previous studies (FS1-FS4).

Finally, relating to the overall autonomic nervous system response to pain, Table 4 displays the interaction effects between autonomic parameters with the heat-pain stimulus quantified by effect size.

**Table 4:** Relationships between heat-pain intensity and features derived from zEMG, cEMG, tEMG, SCL, and ECG.

**Feature**	**zEMG**	**cEMG**	**tEMG**	**SCL**	**ECG**
PK	↑↑	↑↑	↑	↑	na
P2P	↑↑	↑↑	↑	↑↑	na
RMS	↑↑	↑	↑	↑	na
TMNP	↑↑	−	↑	↑	na
TMNV	↓↓	−	↓	↑	na
MAV	↑↑	↑	↑	↑	na
HOMAV1	↑↑	↑	↑	↑	na
HOMAV1n	↑	↑↑	↑	↓	na
HOMAV2	↑↑	↑	↑	−	na
HOMAV2n	↑	↑↑	↑	↓	na
VAR	↑	−	↑	↑	na
SD	↑↑	↑	↑	↑↑	na
R	↑↑	↑↑	↑	↑↑	na
IQR	↑	−	↑	↑↑	na
MD	−	↑	−	−	na
MMNDS	↑	↑	−	−	na
IDS	↓	−	−	−	na
MIDS	−	−	−	−	na
SDMN	↑	↑	−	↑↑	na
SDSD	↑↑	↑*↑↑*	↑	↑↑	na
ApEn	↑	↑	↑	↓	na
FuzzyEn	−	−	−	↓	na
SampEn	−	−	−	↓	na
ShannonEn	↑↑	↑↑	↑	↑	na
SpectralEn	↑	−	−	−	na
PLDF	−	−	−	−	na
LDF	↓	↓	−	−	na
MDCOH	−	−	−	−	na
MNCOH	−	↓	↓	−	na
MMNCOH	−	−	−	−	na
MICOH	−	↓	↓	−	na
CC	↓↓	↓*↓↓*	↓*↓↓*	↓	na
MI	↑↑	↑↑	↑	↑	na
MNF	↑	↑	−	−	na
MDF	↑	↑	−	−	na
ZC	↑↑	↑*↑↑*	↑	na	na
MOF	↑	↑	−	na	na
BW	−	−	−	na	na
CF	−	−	−	na	na
MNRR	na	na	na	na	↓
RMSSD	na	na	na	na	↓
slopeRR	na	na	na	na	↓
**Symbol**	***p*****-value**		**Effect size**		**Interaction**
↓*↓↓*	*p* < 0.05		*d* > 0.8		μ_*B*_>μ_*T*4_
↓↓	*p* < 0.05		0.5 < *d* < 0.8		μ_*B*_>μ_*T*4_
↓	*p* < 0.05		0.2 < *d* < 0.5		μ_*B*_>μ_*T*4_
-	*p* > 0.05		*d* < 0.2		na
↑	*p* < 0.05		0.2 < *d* < 0.5		μ_*T*4_>μ_*B*_
↑↑	*p* < 0.05		0.5 < *d* < 0.8		μ_*T*4_>μ_*B*_
↑*↑↑*	*p* < 0.05		*d* > 0.8		μ_*T*4_>μ_*B*_

*Relationships were quantized into seven levels dependent on their statistical and substantive significance (as shown in the right sub-table)*.

## 4. Discussion

### 4.1. Purpose 1: Identification of General Discriminative Feature Sets

The first purpose of this study was to obtain meaningful, discriminatory sets of generalizable features that capable of high pain recognition rates with minimal bias to the feature selection and classification methods selected. Throughout this investigation to identify meaningful features, several issues were found that are worth discussion.

#### 4.1.1. Feature Selection Approaches

The classification accuracies obtained from the SFS and UFS protocols during the 100 epoch selection served as a robust performance metric for evaluating the modalities and features explored in this study over four classification problems (Tables 2, 3). Assessment of feature sets for the classification of pain threshold (B vs. T1) yielded accuracies of 79.4 and 80.3% for UFS and SFS, respectively. Conversely, when classifying pain tolerance (B vs. T4; the largest stimulus difference) the accuracies for UFS and SFS were found to be 72.9 and 90.3%. This large (and counterintuitive) discrepancy in the UFS results (79.4 vs. 72.9%) was a result of an early local maximum (reaching stop criteria) found during UFS *post-hoc* analysis. For the multi-level three-and five-class problems, accuracies for the UFS chosen feature sets were 63.2 and 40.6%, respectively; whereas SFS selected feature sets with accuracies of 70.3 and 41.4%, respectively. The performance of SFS feature sets has significantly improved as compared to the previous state-of-the-art (Figure 2), and they also required fewer features than the state-of-the-art feature set (e.g., FS4), making them more attractive options from a processing and memory standpoint. However, it should be noted that these accuracies may remain inadequate for clinical or commercial application. Future research with major adjustments is required, whether through novel methods of pre-processing, the discovery of new features or sensing modalities, or the investigation of a more meaningful categorization of pain thresholds. One such example for future research is the application of deep learning techniques that may be able to decipher ambiguous class boundaries using an adequately large dataset.

Specifically, across all classification problems and CVs, the robust feature sets derived in this work (FSa-FSd) significantly outperformed those of Walter et al. (2014), Kächele et al. (2015), and Kächele et al. (2017). The discrepancy between the SFS and UFS feature sets can be attributed to differences in sensitivity to correlation between features. This insensitivity resulted in changes in model accuracy between iterations that are atypical to the standard diminishing trend seen with SFS. For example, as seen in Table 2, the feature selected at *i* = 4 of the pain tolerance problem via UFS—cCC—described less across-class variance than cP2P and cPK features (as indicated by a lower *F*-value). Nevertheless, it still provided useful information that was independent from the previously included features, as indicated by the 12% increase in accuracy (72–84%). Conversely, the information provided by cP2P and cPK, as selected in *i* = 2 and *i* = 3 of the pain tolerance problem, were highly related, resulting in no improvement in accuracy (see the SAP cluster of cEMG in Figure 4). The undesired effects of feature correlation were less profound when using SFS due to the use of Naïve Bayes classification and its aversion to correlated features. Within the complementary feature sets, FS1 used UFS (*F*-value criteria) whereas FS2-FS4 used SFS.

In summary, an advantage of UFS is the direct applicability of findings to the general classification task. The alternative, using a classifier in the feature selection stage, introduces system optimization tailored to that specific classifier and ambiguates the results. A disadvantage of UFS, however, is that the correlation between features is not accounted for in the process of feature selection. This can be somewhat mitigated through the use of an upper threshold on allowable correlation between added features; however, the determination of the optimal threshold for this classification problem is nontrivial. To alleviate this source of ambiguity, no correlation thresholding was used in this work. The purpose of UFS was to verify the discriminative power of each feature in isolation, whereas SFS assesses discriminative power in the presence and context of other features. Largely, the features selected by UFS had strong correlation to one another, as no restriction for similar features was imposed. In future research, this redundancy between chosen features could be removed by using feature projection techniques like principal component analysis (PCA), t-distributed stochastic neighbor embedding (t-SNE), or other advanced feature selection techniques such as swarm intelligence-based algorithms.

#### 4.1.2. Functional Feature Groups

Combining the originally presented *theoretical* feature groups and the analysis of the composition of the topological feature chart (Figure 4), the available features could be categorized into five *functional* feature groups:

1. *Signal amplitude and power (SAP)* was composed of most theoretical amplitude features across EMG and SCL modalities (e.g., RMS, MAV, PK), with the inclusion of theoretical variability features (e.g., VAR, IQR, R) for EMG modalities.
2. *Nonlinear complexity (NLC)* was composed of most measures of information (e.g., ApEn, SampEn, MI) in addition to features that describe signal complexity (e.g., ZC).
3. *Frequency information (FI)* was primarily composed of frequency domain features from the EMG modalities (e.g., MDF, MNF), but also included features that characterize spectral content (e.g., SpectralEn).
4. *Unique (UNI)* was comprised of features from the similarity, stationarity, and linearity theoretical feature groups.
5. *Connecting (CON)* consisted of features that bridge adjacent groups.

As identified by the map, the SAP nodes corresponded to low *k*-NN distances, signifying a high measure of linear and nonlinear correlation between contained features. Differences between feature sets can therefore be seen as minimal when the SAP features are interchanged. Alternatively, the NLC and UNI functional feature groups correspond to medium and high *k*-NN distances, respectively; this signifies that features within these nodes are less correlated to one another. In the NLC functional feature group, the medium range *k*-NN distance allowed for features that better describe class-discriminative information, thus were selected more often than other features within the group (i.e., cShannonEn). However, the performance margin among the alternative features is not so large that perturbations in the dataset may not motivate selection of the other features. In the UNI functional feature group, the repeated appearance of several features across all feature sets (e.g., sSDSD, zCC, cCC, tCC, and hslopeRR) illustrates the variability of class discriminative information present under high *k*-NN distances. In future work, this framework could be applied to quickly validate and rationalize the use of newly proposed features.

In general, functional feature groups were empirically determined collections of features that were grouped into the same node based on the type of information they described. The benefit of using functional feature groups over theoretical feature groups is that associations can be defined between features based on what information they actually contribute rather than their designed purpose. The inclusion of variability features is an example of grouping features based on the information they contribute. As an EMG signal characteristically exhibits zero-mean behavior when the sampling window is sufficiently large, the computation of variability features, such as SD, reduces to simply the signal values, resulting in the variability feature being asymptotically similar to RMS, an amplitude feature. With the current dataset, 7 theoretical feature groups were transformed into 5 functional feature groups that characterize 4 types of information. With this understanding, new features could be designed specifically to improve a particular functional feature group or to define a new functional feature group altogether. One example, based on pre-processing, could be to segment the EMG signal into frequency bands prior to the extraction of features (Koelstra et al., 2012; Abadi et al., 2015). Alternatively, time-frequency representations, which have not yet been exhaustively explored, have successfully been used to improve EMG classification accuracies in other related fields (Englehart et al., 2001; Phinyomark et al., 2011). For any new candidate feature to meaningfully impact the classification problem, it will have to outperform current features within an existing functional group or, better, drive the creation of a new functional group. The latter, in particular, could be achieved through the design of new features, or the introduction of new sensing modalities.

It is important to note that these functional feature groups represent distinct units of information that are determined through mathematics that prioritize generalizability; therefore, they are expected to represent the actual phenomenon and not simply the chosen dataset. In a study by Phinyomark et al. (2017), the same functional groups of EMG features for classification of hand and wrist gestures were identified using multiple datasets with different subjects, experiments, and data acquisition systems. In other words, the topological feature charts are robust and generalize well across multiple datasets, when compared with purely data-driven feature selection techniques. It is reasonable to expect that the functional feature groups defined here should be more applicable to other related pain datasets than those previously proposed in the literature. Moreover, the approach described here can be directly generalized to any other type of data in the field of emotion recognition. Future studies investigating multiple datasets containing data from multiple emotions would be a valuable addition to the literature.

#### 4.1.3. General Discriminative Features

The use of the one-hundred-epoch feature selection approach was intended to be the foundation for generalization of discriminatory information among results highlighted within literature. The framework constructed using the topological network allowed for a thorough comparison of the feature sets determined here, FSa-FSd, and those previously proposed in the literature, FS1-FS4. Although the feature sets determined here through feature selection differed from those previously identified in the literature (despite using an identical dataset to that used in FS4), there were some commonalities. Both the current and previous studies favored the SAP features (particularly from cEMG), and a subset of features within the UNI functional feature group.

Specifically, the mixed modality UNI functional feature group had feature candidates that captured similarity information (i.e., CC) that were consistently selected throughout the feature sets they were available (i.e., FS4, FSa-d). In particular, the CC features involved computing a statistical difference in a physiological signal between a pain-free state (from a baseline measure) and an unknown state measurement window. In a study of Yang et al. (2018), arousal and valence classification was also improved when features were normalized using the difference between an annotation segment and the precedent before, or a neutral state baseline segment. Through this adaptive normalization process, the interactions between dependent variables (autonomic parameters) and random variable (between-subject effect, between trial effect) are minimized, resulting in a direct measurable relationship between the dependent variable and independent variable (pain). Put another way, by normalizing the autonomic response to the subject and monitoring the evolution of autonomic parameters, the fidelity of the system was greatly enhanced. In future works, the relationship between higher-order representations of autonomic parameters and emotion should be explored to characterize the dynamic, transient, and temporally encoded aspects of emotion which have traditionally been ignored.

Through the grouping of features into nodes and their connections, information that is distinct to, and shared between, modalities can be distinguished. From the grouping of the three EMG modalities into their own distinct SAL and NLC nodes, it can be ascertained that each EMG site provides distinct information. This importantly validates the use of these three muscle sites as they each contribute distinct, class-discriminative information. Additionally, SCL forms distinct nodes for SAL, NLC, and UNI functional group. This distinct clustering, in addition to the isolation of a prevalently selected feature, sSDSD, validates the use of the SCL as a sensing modality. In contrast to other modalities, ECG does not occupy a node by itself. This signifies that of all the modalities evaluated here, ECG contributed the least distinct information. Though hslopeRR was selected in multiple feature sets, it is possible that features from other modalities could be used as a comparable replacement, thus eliminating the need for this modality. In future work, there remains great potential in the exploration of additional modalities that could augment the current feature space (e.g., EEG, MEG, and fMRI, intramuscular EMG). Though each new candidate modality will have its drawbacks, whether complexity, invasiveness or cost, their contribution could be validated by including them in such an analysis.

In summary, one could expect a feature set involving several EMG and SCL features extracted from the SAP and UNI functional feature groups to be sufficient to represent the targeted emotion pain classes. It should be noted that the variability among features in these sets with comparable classification accuracies signifies that no one feature set should be blindly adopted at the cost of abandonment of other equally viable feature sets. One should exercise caution when presenting one local optimum feature set as the best, or otherwise risk the loss of equally useful features. That is, there is sufficient redundancy between the features and modalities to provide the designer with some flexibility. The understanding provided by the topological feature charts could enable emotion recognition system designers to incorporate additional prior knowledge, leading to more robust and generalizable feature and modality selection.

### 4.2. Purpose 2: Insights Into Autonomic Parameters

The second objective of this study was to quantify and explain the associations between these autonomic parameters (or features) to improve comprehension of the actual classification problem. Through the combination of feature-pain interaction effects and functional feature groups defined by the topological simplification, autonomic nervous system activity was observed.

Largely, the SAP functional feature group for the facial muscles (zEMG-cEMG) and the trapezius had a moderate-high, and weak strength positive interaction with pain (Table 4). This interaction effect supports established theories that electrical muscle activity of myofacial muscles have the ability to detect sympathetic arousal (Nilges and Traue, 2007). Additionally, the ability to detect facial expressions in response to pain are corroborated by earlier facial expression recognition studies that achieved great success (Hamedi et al., 2018). In general, the high interaction effect between cEMG and pain intensity in the context of a high arousal, neutral valence stimulus is consistent with expectations from facial expression studies (Lee et al., 2009; AlZoubi et al., 2012; Khezri et al., 2015). Furthermore, the SAP functional feature group for the trapezius muscle similarly had a positive interaction effect with pain-intensity; however, the strength was weaker than those of facial EMG. Nevertheless, tEMG has successfully been harnessed as an unconscious marker of stress in the context of driver induced stress (Wijsman et al., 2013), corroborating its interaction effect with pain in this study. Therefore, the interaction effect between tEMG SAP and pain supports evidence of an increase in muscle tone as a stress response to painful stimuli, and reflectory head movements. The relationship between an increase in SAP of EMG signals in response to a heightened state of arousal is also consistent with the literature Kim and André (2008). These findings, however, are inconsistent with the implications of studies using the popular Database for Emotion Analysis Using Physiological Signals (DEAP), where facial EMG signals have been found to provide no meaningful contribution for the classification of emotions defined using the arousal-valence dimensions (Koelstra et al., 2012). Further investigation is thus needed using multiple emotion datasets to determine the optimal conditions for implementation of facial EMG in this context. Importantly, facial EMG within emotion datasets are typically collected using lower sampling frequencies than other EMG applications, which could alias key components of the signal that provide benefit in positive exemplars of facial EMG implementation.

Another source of discriminative information lies within the NLC functional feature group of facial EMG. Specifically, cZC and zZC, respectively, were found to have strong and moderate positive interaction effects with pain. Increases in ZC correspond to an increase in muscle tone due to sympathetic activation of the muscles. In a similar relationship to that of SAP, the interaction effect between the NLC function group for the facial muscles and the trapezius with pain were of moderate-high, and weak strength, respectively.

The strong interaction effect found between the EMG CC features and pain indicated that there was a repeatable autonomic excitation that evolved in response to pain. This could reinforce the earlier notion that sympathetic responses to painful stimuli are influenced by your current mental state. Potentially due to repeated stimuli over the course of the experiment or a gradual change in emotional-state, the response to painful stimuli later in the experiments elicited subtle differences as compared to earlier recordings of the same level. The differences led to higher reliability of EMG CC features as compared to SAP features when characterizing this response.

Interestingly, the topological clustering segmented SCL amplitude feature into two categories, one with only amplitude features (identified as SAP functional group) and the other comprised of an assortment of amplitude, frequency, linearity, entropy, and similarity features (identified as UNI functional group). The SCL SAP functional group had a weak strength positive interaction with pain intensity. The SCL UNI functional group had a moderate strength positive interaction with pain intensity and justifies its common use as an indicator of arousal-state. Specifically, features within this UNI cluster were capable of capturing the phasic/transient activity of SCL, i.e., sP2P, sR, and sSDSD features, whereas features that were within the SAP SCL group were able to capture tonic activity of SCL, i.e., sRMS and sMAV (Bach et al., 2010).

While metrics extracted from ECG (i.e., heart-rate, blood pressure, heart-rate variability, and blood oxygen level) are conventionally used alongside RR by anesthesiologist to monitor pain levels and medical intervention effectiveness for surgical procedures, the interaction effect between pain level and ECG during this study was found to be weak and negative. This result suggests that the onset of pain results in an observable parasympathetic reaction on the heart, while all other modalities elicited a sympathetic reaction. The observation is likely misleading to the true nature of the autonomic response to pain. Likely, as a reflex to sudden pain, the subjects exhaled sharply overpowering the sympathetic rise of heart-rate with a behavior-induced reduction of heart-rate caused by respiratory sinus arrhythmia. While this phenomenon would explain the nature of this relationship, respiration rate is not present in the current dataset; thus, future work is required for a definitive conclusion.

The emotional state elicited in response to pain stimuli resembles that of imminent threat fear or suspense characterized by the positive relationship with corrugator SAP, positive relationship with phasic SCL, and negative relationship with heart-rate features (Hubert and de Jong-Meyer, 1991; Kreibig, 2010). Both fear and suspense emotional-states manifest in high arousal and neutral valence dimensions that are related to activity in noradrenergic structures. During the onset of pain, these structures produce norepinephrine, the neuropeptide responsible for broadcasting a sympathetic response to the autonomic nervous system. This sympathetic response manifests in a heightened sense of arousal characterized by phasic SCL activity, facial expression, and a sudden increase in heart rate. The overall observed parasympathic response for the heart was likely forced by either one of two factors. The first factor was respiratory sinus arrhythmia, as discussed in the prior paragraph. The second factor could be due to a descending nociceptive pathway involving the activation of opiodergic structures, the periaqueductal gray, that modulated the perceived pain (Pavlovic and Bodnar, 1998). The release of these neuropeptides envoke an autonomic response to inhibit signaling of affected second-order neurons and reduce sympathetic tone resulting in lower heart rate and respiration rate. These endocrine signals governed by neurological processes and behavioral reactions were responsible for the observable SCL, EMG, and ECG embodiments that resembled emotional-states within this, and other pain related studies.

## 5. Conclusion

In summary, the two objectives sought out through this study were completed. The first purpose was an exploration to obtain a general understanding of effective autonomic parameters and isolate a robust feature set for each classification problem. The feature sets provided through this protocol contained fewer features and improved performance compared to the state-of-the-art. The second purpose was to form associations between autonomic parameters through topological data analysis. The relationship between the features chosen within our selection and those of four accompanying studies provided insight into the types of information necessary for the development of a pain detection system. Of note, the signal amplitude and power, and unique functional feature groups of EMG modalities provided useful information across all feature sets, while nonlinear complexity information appeared often.

The framework constructed here to define the information contribution of features also provided an environment to evaluate future contributions to this field. This approach can be directly generalized to any other type of emotion data. Directions for future work highlighted by this study include the introduction of new modalities, new feature types, new feature selection techniques, and new feature projection techniques, each of which can be evaluated using the described topological network.

## Ethics Statement

The dataset outlined by Gruss et al. (2015) was adopted for this work as it was publicly available and includes physiological modalities common across a majority of related works. The collection of the data, as explained by Walter et al. (2013), was carried out in accordance with the recommendations of the ethics committee of the University of Ulm with written informed consent from all subjects. All subjects gave written informed consent in accordance with the Declaration of Helsinki. The protocol was approved by the ethics committee of the University of Ulm (196/10-UBB/bal). The data include surface EMG from the zygomaticus (zEMG), corrugator (cEMG), and trapezius (tEMG) muscles, ECG, and SCL elicited in response to a pain-inducing heat stimulus. For each trial, a randomly selected pain stimulus level was applied for 4 s and the physiological response was recorded for 5.5 s. Eighty-five participants conducted 20 trials at each of the five pain-intensity levels.

## Author Contributions

The study design, analysis and writing were conducted in collaboration by EC, AP, and ES.

### Conflict of Interest Statement

The authors declare that the research was conducted in the absence of any commercial or financial relationships that could be construed as a potential conflict of interest.
